# A randomised clinical trial of two docetaxel regimens (weekly *vs* 3 week) in the second-line treatment of non-small-cell lung cancer. The DISTAL 01 study

**DOI:** 10.1038/sj.bjc.6602241

**Published:** 2004-11-23

**Authors:** C Gridelli, C Gallo, M Di Maio, E Barletta, A Illiano, P Maione, S Salvagni, F V Piantedosi, G Palazzolo, O Caffo, A Ceribelli, A Falcone, P Mazzanti, L Brancaccio, M A Capuano, L Isa, S Barbera, F Perrone

**Affiliations:** 1 Oncologia Medica, Azienda Ospedaliera S Giuseppe Moscati, Avellino; 2 Statistica Medica, Seconda Università di Napoli; 3 Unità Operativa Sperimentazioni Cliniche, Istituto Nazionale Tumori, Napoli; 4 Oncologia Medica B, Istituto Nazionale Tumori, Napoli; 5 Pneumooncologia, II Divisione, Ospedale Monaldi, Napoli; 6 Oncologia, Ospedale Regionale, Parma; 7 Pneumooncologia, I Divisione, Ospedale Monaldi, Napoli; 8 Oncologia Medica, ULSS 15 Regione Veneto; 9 Oncologia Medica, Ospedale S Chiara, Trento; 10 Oncologia Medica A, Istituto Regina Elena, Roma; 11 Oncologia, Ospedali Riuniti, Livorno; 12 Oncologia Medica, Azienda Ospedaliera Umberto I, Torrette di Ancona; 13 Chirurgia Toracica, Ospedali Riuniti, Foggia; 14 Oncologia Medica, Ospedale Serbelloni, Gorgonzola; 15 Pneumologia, Ospedale Mariano Santo, Cosenza

**Keywords:** quality of life, docetaxel, weekly schedule, second line, advanced NSCLC

## Abstract

Docetaxel (75 mg m^−2^ 3-weekly) is standard second-line treatment in advanced non-small-cell lung cancer (NSCLC) with significant toxicity. To verify whether a weekly schedule (33.3 mg m^−2^ for 6 weeks) improved quality of life (QoL), a phase III study was performed with 220 advanced NSCLC patients, ⩽75 years, ECOG PS ⩽2. QoL was assessed by EORTC questionnaires and the Daily Diary Card (DDC). No difference was found in global QoL scores at 3 weeks. Pain, cough and hair loss significantly favoured the weekly schedule, while diarrhoea was worse. DDC analysis showed that loss of appetite and overall condition were significantly worse in the 3-week arm in the first week, while nausea and loss of appetite were more severe in the weekly arm in the third week. Response rate and survival were similar, hazard ratio of death in the weekly arm being 1.04 (95% CI 0.77–1.39). A 3-weekly docetaxel was more toxic for leukopenia, neutropenia, febrile neutropenia and hair loss; any grade 3–4 haematologic toxicity was significantly more frequent in the standard arm (25 *vs* 6%). The weekly schedule could be preferred for patients candidate to receive docetaxel as second-line treatment for advanced NSCLC, because of some QoL advantages, lower toxicity and no evidence of strikingly different effect on survival.

Platinum-based chemotherapy is the most common treatment for patients with advanced non-small-cell lung cancer (NSCLC), thanks to a small survival advantage found in a metanalysis of 11 randomised trials (Non Small Cell Lung Cancer Collaborative Group, 1995). Cisplatin or carboplatin are usually administered in two-drug combinations with paclitaxel, docetaxel, gemcitabine, or vinorelbine. About one-third of patients achieve clinical remission and another third temporary disease stabilisation; almost all patients ultimately suffer progression of the disease and die for lung cancer.

At the time of disease progression many patients still have a good performance status and a second-line chemotherapy is a reasonable therapeutic option. Docetaxel 75 mg m^−2^ once every 3 weeks prolongs survival compared to best supportive care (Shepherd *et al*, 2000) and produces less deterioration of pain and fatigue (Dancey *et al*, 2004). However, myelosuppression is frequent and severe (Fossella *et al*, 2000; Shepherd *et al*, 2000).

Weekly scheduling of docetaxel (Hainsworth *et al*, 1998) might remarkably reduce myelotoxicity in pretreated NSCLC patients (Lilenbaum *et al*, 2001), without decreasing antitumoral activity. We performed a multicentre randomised clinical trial to compare a weekly schedule of docetaxel against the standard 3-week regimen in terms of quality of life (QoL), toxicity and efficacy.

## PATIENTS AND METHODS

### Patient selection and baseline assessment

Patients, younger than 75 years, were required histological or cytological proof of NSCLC, stage IV or IIIB with malignant pleural effusion and/or metastatic supraclavicular lymphnodes, evidence of progressive disease during or after first-line chemotherapy, ECOG performance status 0–2, adequate haematology (absolute neutrophil count ⩾2000 mm^−3^, platelets ⩾100 000 mm^−3^ and haemoglobin ⩾10 g dl^−1^) and biochemistry (serum creatinine ⩽1.25 × upper normal limit, SGOT and SGPT and bilirubin ⩽1.25 × upper normal limit, unless due to liver metastases), availability to complete QoL questionnaires, written informed consent. Patients with symptomatic brain metastases or prior invasive malignancies were excluded. The protocol was approved by ethical committees at each participating institution.

Complete history and physical examination, routine haematology and biochemistry, staging with chest radiographs, chest, brain and abdominal computed tomography (CT), and QoL assessment were required before randomisation.

### Treatment schedules

The control arm included docetaxel 75 mg m^−2^ on day 1 every 3 weeks for six cycles of chemotherapy; the experimental treatment was docetaxel 33.3 mg m^−2^ on days 1, 8, 15, 22, 29, 36 every 8 weeks (6 weeks of treatment followed by 2 weeks of rest) for two cycles (i.e. 12 administrations); treatment in both arm could be suspended in case of progression or unacceptable toxicity. Planned dose-intensity was the same in both arms (i.e. 25 mg m^−2^ week^−1^). Further therapy was discretional. The following criteria were required to give chemotherapy: neutrophils ⩾1500 *μ*l^−1^, platelets ⩾100 000 *μ*l^−1^, haemoglobin ⩾8 g dl^−1^ and absence of grade ⩾2 nonhaematologic toxicity (excluding alopecia). Lacking these conditions, a 1-week delay was planned and treatment was stopped after two consecutive delays. Dose reductions and prophylactic use of haemopoietic colony stimulating factors were not allowed. Docetaxel was given intravenously in 1-h; dexamethasone (8 mg i.m. or i.v.) was given at −12, 0, +12, +24 and +36 h of every docetaxel administration in the standard arm and at −12, 0 and +12 h in the weekly arm.

### Design

Centralised phone randomisation (1 : 1 ratio) was performed at the Clinical Trials Unit of the National Cancer Institute of Naples, using a computer-driven minimisation procedure stratified by center, performance status (0 *vs* 1 *vs* 2), objective response to first-line chemotherapy (complete or partial response *vs* stable or progressive disease) and type of previous treatment (with *vs* without platinum).

The primary end point of the study was QoL. Three instruments were applied.

**Figure 1:**
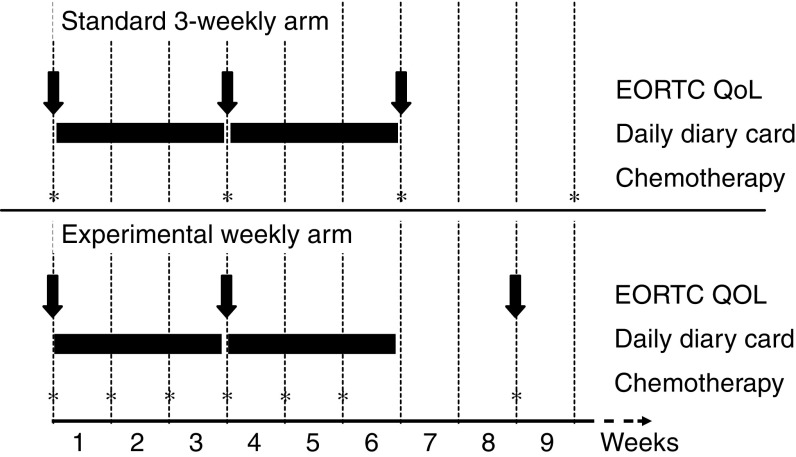
Timing of QoL assessment.

The EORTC QLQ-C30 explores functional scales (physical, role, emotional, social and cognitive functioning) symptoms (fatigue, pain, emesis, dyspnea, insomnia, appetite, diarrhoea, constipation), financial impact, and global health status (Aaronson *et al*, 1993). The EORTC QLQ-LC13 assesses lung cancer symptoms (Bergman *et al*, 1994). Scores were computed according to EORTC rules (Fayers *et al*, 1999). Questionnaires were administered before randomisation and 3 weeks after beginning of therapy in both arms; a third questionnaire was administered before the third cycle in the 3-week arm and before the second cycle in the weekly arm (Figure 1

).

The Daily Diary Card (DDC) was designed by the Medical Research Council Lung Cancer Working Party (Fayers *et al*, 1991; Fayers, 1995) to capture rapid and transient changes of sleeping, mood, well-being, level of activity, nausea, vomiting, appetite loss and pain. DDC was collected after 3 and 6 weeks (Figure 1).

Overall survival was defined as the interval from date of randomisation and date of death or date of last follow-up information for living patients. Objective response, categorised according to RECIST (Therasse *et al*, 2000), was evaluated at the end of the third and sixth cycles of treatment (approximately 9 and 18 weeks) in the standard arm and after six and 12 administrations (approximately 8 and 16 weeks) in the experimental arm. The best response was recorded for each patient and confirmation was not performed. Patients who stopped treatment because of toxicity or refusal or death before restaging were defined as nonresponders in the calculation of response rate. Time to disease progression was not described nor analysed, because of the bias determined by the unequal cycle duration in the two treatment arms (Green *et al*, 2002b).

For toxicity assessment, haematology was repeated weekly and biochemistry at 3 and 6 weeks in both arms. Toxicity was coded according to NCI-CTC (National Cancer Institute – Cancer Therapy Evaluation Program, 1999). The worst degree of toxicity experienced during the treatment was computed for each patient.

### Sample size

Global health status scale (items 29 and 30) of EORTC QLQ-C30 after 3 weeks from the start of chemotherapy was used to plan sample size. A 90% power to detect an effect size of 50% (i.e. a difference between mean scores of global health status equal to 50% of the standard deviation) after 3 weeks of chemotherapy was planned. Such an effect size has been correlated with conditions of ‘moderate’ or ‘very much’ positive changes in a subjective satisfaction questionnaire (Osoba *et al*, 1998). With a two-sided significance level of 0.05, a total of 172 patients were needed (nQuery Advisor® 4.0, Statistical Solutions Ltd, Cork, Ireland). Assuming a 25% dropout rate, 215 patients were required.

### Statistical analysis

All the analyses were based on ‘intention-to-treat’. Statistical tests were limited to QoL data at 3 weeks, because comparison beyond that time could be biased by different scheduling (Figure 1). With EORTC questionnaires, differences from baseline scores were compared by Wilcoxon rank-sum test. QoL response was defined ‘improved’ (3-week score ⩾10 points better than baseline), ‘worse’ (score ⩾10-points worse than baseline), or ‘stable’ in between (Osoba *et al*, 1998). Comparisons of QoL response was carried out with an exact linear rank test. For DDC, the daily rate of patients falling into the two worst scores was calculated for each item. A null hypothesis that the effect of treatments did not change across time was assessed by an interaction test across weeks 1–3 (reported as *P*_int_). The exact Wilcoxon rank-sum test was applied either on the overall period if there was no statistical interaction (reported as *P*_overall_), or separately for each week if a significant interaction was evident (reported as *P*_1_, *P*_2_, and *P*_3_ for weeks 1–3 respectively). All *P*-values were considered significant if ⩽0.05.

Planned survival analysis required 190 deaths to detect a 50% improvement of median survival with 80% power, an expected median survival of 30 weeks in the control arm (Shepherd *et al*, 2000), two-sided alpha level of 0.05, and one interim analysis. The latter, carried out with blinded treatment, using the alpha spending function (Lan and DeMets, 1983), based on O'Brien and Fleming (1979) sequential group design, did not produce study termination. Survival curves were estimated by Kaplan and Meier (1958) method. Cox (1972) model was applied for multivariable analyses. The Fisher's exact test was applied to compare the objective response rate, defined as the proportion of complete and partial responses on the whole number of patients. Toxicities were compared by an exact linear rank test (all grades) and by Fisher's exact test (grade 3–4 *vs* 0–2). East software® 2.0, 1992, Cytel Software Corp, Cambridge, MA, USA and StatXact5.0.3, 2001, Cytel Software Corp., Cambridge, MA, USA were used.

## RESULTS

### Patient characteristics

**Figure 2:**
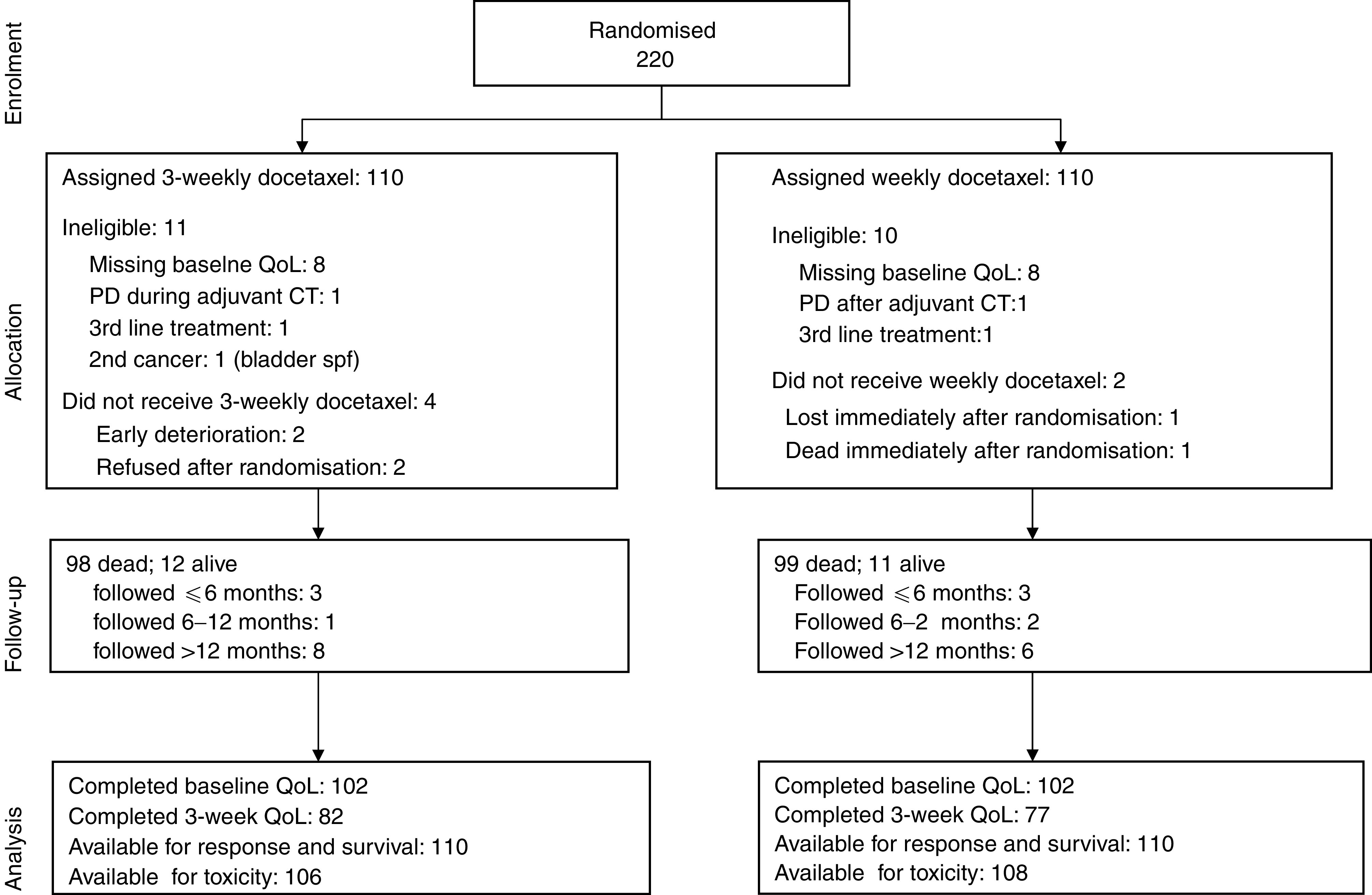
Study flow according to CONSORT.

In total, 220 patients were randomised, between December 2000 and August 2002; 21 were found ineligible after randomisation because had not filled in baseline QoL (16 cases), progressed during adjuvant chemotherapy (two cases) or during second-line chemotherapy (two cases) and one case because of a previous bladder neoplasm (Figure 2

). Baseline characteristics for the 220 patients (Table 1

**Table 1:** Baseline characteristics by treatment arm

**Variable**	**3-weekly (*N*=110)**	**Weekly (*N*=110)**	**Total (*N*=220)**
*Age*			
Median	62	63	63
Range	26–74	28–75	26–75
			
*Sex*			
Males	88 (80%)	95 (86%)	183 (83%)
Females	22 (20%)	15 (14%)	37 (17%)
			
*Stage*			
IIIB	21 (19%)	10 (9%)	31 (14%)
IV	89 (81%)	100 (91%)	189 (86%)
			
*Histotype*			
Squamous/epidermoid	31 (28%)	38 (35%)	69 (31%)
Adenocarcinoma	58 (53%)	50 (45%)	108 (49%)
Large cells	3 (3%)	2 (2%)	5 (2%)
Mixed	3 (3%)	3 (3%)	6 (3%)
Undefined	15 (14%)	17 (15%)	32 (15%)
			
*PS*			
0	35 (32%)	36 (33%)	71 (32%)
1	58 (53%)	56 (51%)	114 (52%)
2	17 (15%)	18 (16%)	35 (16%)
			
*Previous treatment with platinum*			
No	16 (15%)	18 (16%)	34 (15%)
Yes	94 (85%)	92 (84%)	186 (85%)
			
*Response to first line chemotherapy*			
Complete or partial	34 (30%)	36 (32%)	70 (32%)
Stable or progression	76 (70%)	74 (68%)	150 (68%)

) were balanced between the two arms. Median age was 63 years, 83% of patients were male, ECOG performance status was 0–1 in 84 and 86% of subjects had stage IV disease. Previous chemotherapy was platinum-based in 85% of the patients.

### Chemotherapy

After randomisation, six patients did not receive the assigned treatment (Figure 2): in the 3-week arm, two refused treatment, one suffered progression of brain metastases and one had acute clinical deterioration, before starting chemotherapy; in the weekly arm, one patient died and one was lost, immediately after randomisation. In the 3-week arm, 69% of patients received at least half of the planned therapy (three cycles) and 23% completed the treatment; in the weekly arm 62% received half of the planned therapy (six administrations) and 25% completed the treatment. Treatment was stopped because of disease-related causes (progression or death) in 59.1 and 51.8%, and because of toxicity or refusal in 10.9 and 20.9% of the patients in the weekly and the 3-weekly arms, respectively.

### EORTC questionnaires

In the 3-week arm, out of 102 patients with baseline data, 100 were alive at 3 weeks and 82 filled in the second questionnaire, while the third one was completed by 67. In the weekly arm, out of 102 patients with baseline data, 98 were alive at 3 weeks and 77 filled in the second questionnaire, while the third one was completed by 43. At 3 weeks there was no statistical difference in compliance between the two arms (*P*=0.20).

Baseline mean scores were similar between the two arms for all of QoL items (Table 2

**Table 2:** Quality of life analysis

**Domain/item**	**3-weekly docetaxel**				**Weekly docetaxel**				***P*-value**
	**Baseline, mean (s.d.)**	**Improved, *N* (%)**	**Stable, *N* (%)**	**Worse, *N* (%)**	**Baseline, mean (s.d.)**	**Improved, *N* (%)**	**Stable, *N* (%)**	**Worse, *N* (%)**	
Global QoL	55.2 (21.2)	23 (28)	35 (43)	24 (29)	55.5 (24.8)	16 (21)	42 (55)	19 (25)	0.90
Physical	72.6 (20.7)	11 (14)	50 (62)	20 (25)	69.4 (21.4)	12 (16)	54 (72)	9 (12)	0.09
Role	70.4 (31.1)	21 (27)	33 (42)	25 (32)	64.3 (33.1)	23 (31)	35 (47)	16 (22)	0.26
Emotional	71.0 (19.2)	14 (17)	46 (56)	22 (27)	69.6 (21.6)	17 (22)	47 (61)	13 (17)	0.14
Cognitive	87.7 (17.7)	13 (16)	46 (56)	23 (28)	86.4 (16.9)	16 (21)	48 (62)	13 (17)	0.11
Social	79.6 (23.7)	19 (23)	38 (46)	25 (30)	77.1 (24.1)	21 (28)	40 (53)	15 (20)	0.22
Pain	28.9 (27.4)	23 (28)	36 (44)	22 (27)	30.7 (31.7)	29 (38)	32 (42)	16 (21)	0.20
Appetite loss	15.5 (23.8)	9 (11)	58 (72)	14 (17)	23.5 (27.2)	16 (21)	44 (58)	16 (21)	0.60
Constipation	28.7 (29.8)	23 (28)	48 (59)	10 (12)	21.5 (27.3)	19 (25)	50 (66)	7 (9)	0.93
Financiary	17.8 (28.3)	9 (11)	58 (72)	13 (16)	13.9 (22.2)	9 (12)	59 (78)	8 (11)	0.41
Fatigue	32.9 (20.6)	15 (19)	32 (40)	34 (42)	40.0 (25.9)	19 (25)	29 (38)	29 (38)	0.42
Nausea/vomiting	6.1 (14.3)	13 (16)	58 (72)	10 (12)	7.7 (16.4)	11 (14)	49 (64)	17 (22)	0.18
Sleeping	28.7 (30.2)	18 (22)	45 (56)	17 (21)	28.7 (31.3)	12 (16)	40 (53)	23 (31)	0.14
Diarrhoea	3.7 (14.9)	3 (4)	62 (78)	14 (18)	4.4 (13.2)	5 (7)	47 (64)	22 (30)	0.22
Dyspnoea	26.5 (18.1)	11 (14)	55 (68)	15 (19)	27.1 (19.9)	11 (14)	51 (67)	14 (18)	0.95
Cough	37.3 (24.2)	14 (17)	50 (62)	17 (21)	38.7 (26.8)	27 (36)	40 (53)	9 (12)	**0.007**
Haemoptysis	5.0 (13.7)	3 (4)	76 (95)	1 (1)	3.3 (10.1)	4 (5)	69 (92)	2 (3)	0.95
Sore mouth	8.9 (18.2)	6 (7)	63 (78)	12 (15)	6.3 (15.5)	4 (5)	53 (71)	18 (24)	0.16
Swallowing	8.3 (17.3)	11 (14)	63 (78)	7 (9)	7 (17.3)	7 (10)	57 (77)	10 (14)	0.27
Neuropathy	20.5 (27.2)	9 (12)	56 (72)	13 (17)	19.7 (28.1)	10 (13)	53 (70)	13 (17)	0.95
Hair loss	14.2 (25.5)	5 (6)	30 (37)	46 (57)	13.3 (23.2)	8 (11)	54 (72)	13 (17)	**<0.0001**
Pain chest	17.2 (23.4)	12 (15)	62 (77)	7 (9)	16.3 (25.3)	10 (14)	53 (73)	10 (14)	0.52
Pain shoulder	26.1 (29.3)	17 (21)	50 (62)	14 (17)	25.0 (28.6)	20 (27)	47 (63)	8 (11)	0.24
Pain elsewhere	22.6 (26.4)	23 (29)	41 (52)	15 (19)	21.5 (27.5)	17 (23)	52 (70)	5 (7)	0.64

**Figure 3:**
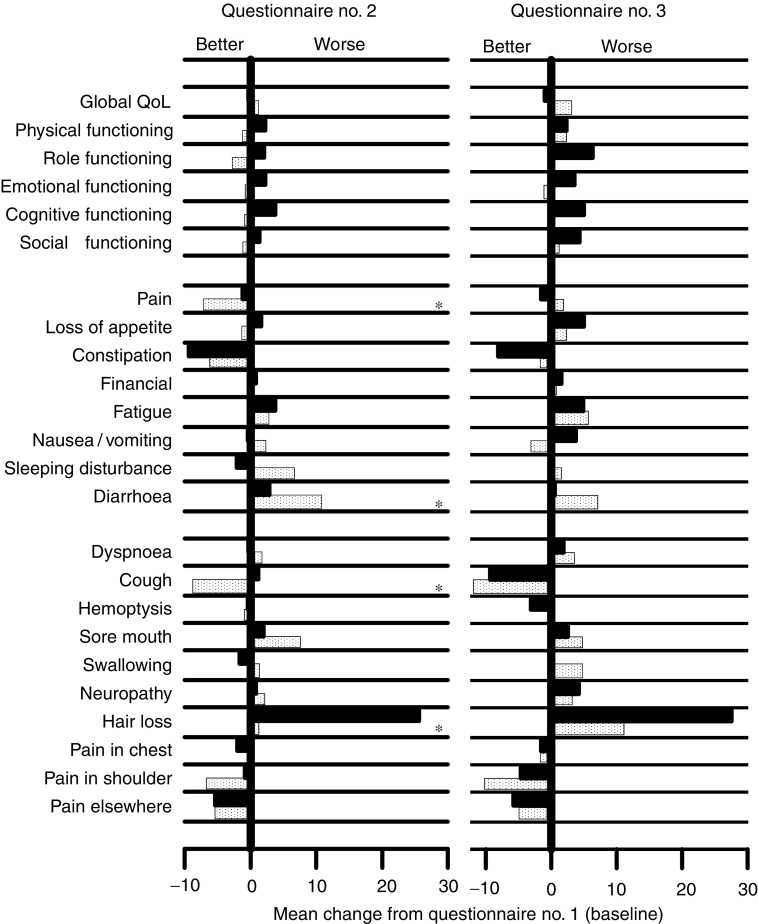
Mean change in EORTC quality of life scores at 3 weeks and at 6 weeks from baseline. Dotted bars represent weekly docetaxel, black bars represent standard 3-weekly docetaxel. Asterisks indicate statistically significant differences.

Bold values are statistically significant.

). Mean changes from baseline of QoL domains are displayed in Figure 3

. Global QoL did not show significant variations. Functioning scales did not change much during the first 3 weeks, while varied more evidently at the third questionnaire; in all cases, scores were consistently better with weekly docetaxel (dotted bars). For patients in the weekly arm, statistically significant differences at the second questionnaire were observed for better pain (*P*=0.04) and cough (*P*=0.007), less hair loss (*P*<0.001) and worse diarrhoea (*P*=0.01). QoL response after 3 weeks showed significant differences only for cough and hair loss, in favour of patients in the weekly arm (Table 2).

### Daily Diary Card

**Figure 4:**
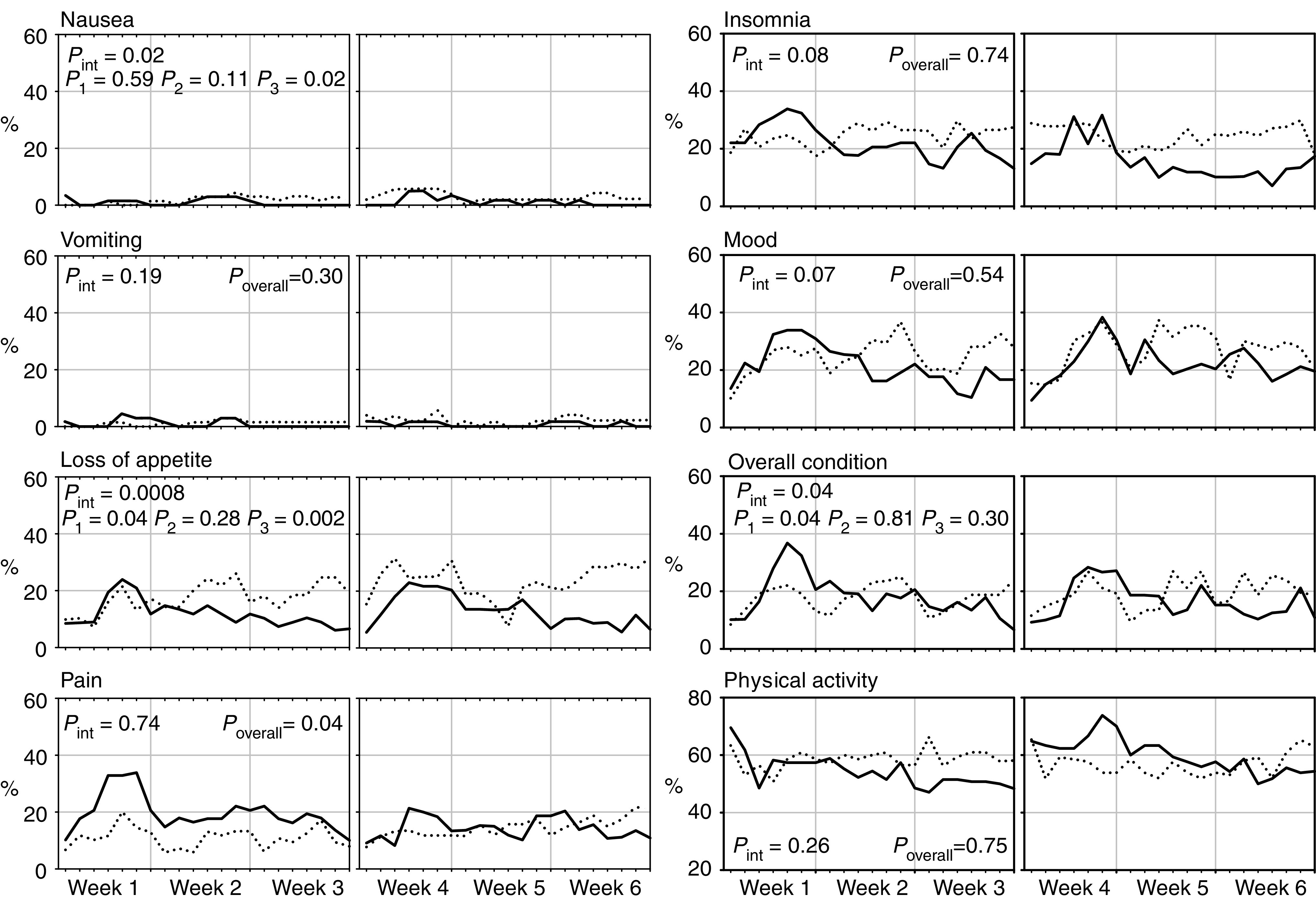
Percent of patients falling into the two worst scores day-by-day for each item of the Daily Diary Card. Solid line=3-weekly docetaxel. Dotted line=weekly docetaxel. *P*_int_=test for interaction across weeks; *P*_1_, *P*_2_, *P*_3_=Wilcoxon rank-sum test for week 1, 2 and 3 respectively; *P*_overall_=Wilcoxon rank-sum test for overall period of weeks 1–3.

DDC was compiled and delivered by 69 and 61 patients in the 3-weekly arm and by 70 and 56 in the weekly arm, after 3 and 6 weeks, respectively. The rates of patients falling into the two worst categories for each item day by day (Figure 4

) show that nausea and vomiting had negligible impact in both arms, while with weekly treatment loss of appetite tended to be more evident, and pain was better controlled during the first 3 weeks. For vomiting, pain, insomnia, mood and physical activity there was no evidence that the effect of treatments changed across the time (*P*_int_>0.05); for all of these items no significant differences between arms were observed (*P*_overall_>0.05), except for pain that was constantly lower in the weekly arm throughout the whole period of observation (*P*_overall_=0.04). On the other hand, the interaction test was statistically significant (*P*_int_⩽0.05) for nausea, loss of appetite and overall condition, suggesting that the effect of treatments on QoL changed across weeks. Indeed, separate comparisons by weeks showed that, in the 3-week arm, loss of appetite and deterioration of general condition were worse during the first week (*P*_1_=0.04 for both), while, in the weekly arm, nausea and loss of appetite were more severe during the third week (*P*_3_=0.02 and 0.002, respectively).

### Efficacy

**Figure 5:**
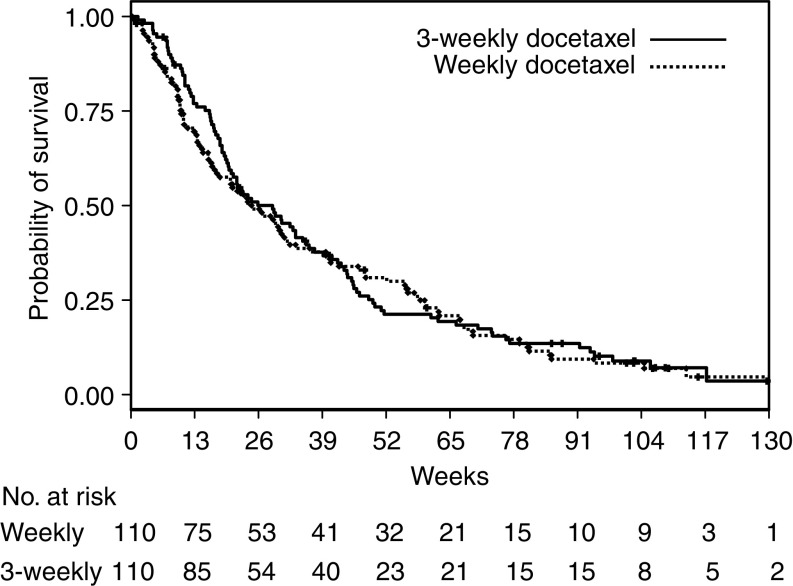
Survival curves estimated by the Kaplan–Meier method. Vertical signs refer to censored patients.

All 220 randomised patients were included in intention-to-treat analyses, irrespective of whether they received protocol therapy. With 197 deaths, 98 in the standard arm and 99 in the experimental arm, the hazard ratio of death was 1.04 (95% CI 0.77–1.39; *P*=0.80) for patients receiving weekly docetaxel, in a Cox model including performance status, age, sex, stage, previous cisplatin and response to first-line treatment as covariates. Overall survival curves are shown in Figure 5

. Median survival was 29 weeks (95% CI 21–36) and 25 weeks (95% CI 18–34), while 1-year survival probability was 0.21 and 0.31, in the 3-week and weekly arm, respectively. Only partial responses were observed with an objective response rate of 2.7 and 5.5% in the 3-weekly and weekly arm, respectively (*P*=0.50).

### Toxicity

Patients who received at least one dose of chemotherapy (*N*=214) were included in toxicity analysis (Table 3

**Table 3:** Toxicity analysis

	**Worst NCI-CTC grade (%)**													
	**3-weekly docetaxel**						**Weekly docetaxel**							
**Toxicity**	**0**	**1**	**2**	**3**	**4**	**5**	**0**	**1**	**2**	**3**	**4**	**5**	***P***^a^	***P***^b^
Anaemia	74	12	11	2	1	—	85	8	7	—	—	—	0.03	0.12
Leukopenia	71	6	14	7	3	—	94	1	3	2	—	—	**<0.0001**	**0.02**
Neutropenia	71	2	8	8	11	—	91	6	2	1	1	—	**0.0001**	**<0.0001**
Febrile neutropenia	95	—	—	4	1	—	100	—	—	—	—	—	0.06	**0.03**
Neutropenic infection	97	—	—	1	1	1	100	—	—	—	—	—	0.24	0.12
Non-neutropenic infection	99	—	1	—	—	—	93	1	3	3	1	—	**0.02**	0.12
Platelets	97	1	1	—	1	—	97	—	2	—	1	—	0.99	0.99
Platelet transfusion	99	—	—	1	—	—	100	—	—	—	—	—	NA	0.50
RBC transfusion	95	—	—	5	—	—	99	—	—	1	—	—	NA	0.12
Allergy	98	—	—	2	—	—	97	1	1	1	—	—	0.99	0.62
Fatigue	44	29	20	4	3	—	46	30	19	6	—	—	0.68	0.78
Heart rhythm	97	—	1	1	—	1	98	—	—	2	—		0.79	0.99
Heart general	97	—	—	2	—	1	96	1	—	—	—	3	0.67	0.99
Fever	92	7	1	—	—	—	91	7	2	1	—	—	0.59	0.99
Weight loss	93	5	1	1	—	—	93	3	5	—	—	—	0.71	0.50
Hair loss	64	17	20	—	—	—	80	14	7	—	—	—	**0.004**	—
Local react.	99	—	1	—	—	—	98	1	1	—	—	—	0.99	—
Skin	92	5	3	1	—	—	94	4	2	—	—	—	0.39	0.50
Anorexia	76	20	4	1	—	—	80	14	6	—	—	—	0.54	0.50
Constipation	85	11	4	—	—	—	92	7	1	—	—	—	0.12	—
Diarrhoea	79	11	7	2	1	—	74	14	9	3	—	—	0.50	0.99
Nausea	81	13	6	—	—	—	79	17	5	—	—	—	0.75	—
Vomiting	93	4	4	—	—	—	87	7	6	—	—	—	0.25	—
Stomatitis	83	9	7	1	—	—	87	9	4	—	—	—	0.36	0.50
Liver	94	1	3	1	1	—	95	3	2	—	—	—	0.56	0.24
Motor Neurop.	95	2	2	1	—	—	95	2	1	2	—	—	0.99	0.99
Sensit. Neurop.	81	12	7	—	—	—	81	13	5	2	—	—	0.91	0.50
Kidney	99	—	1	—	—	—	99	1	—	—	—	—	0.99	—

a All grades (exact Wilcoxon rank-sum test).

b Grades 3–4–5 *vs* grades 0–1–2 (Fisher's exact test); RBC=red blood cells; NA=not assessable.

Bold values are statistically significant.

). Standard 3-week docetaxel caused more leukopenia, neutropenia, febrile neutropenia and hair loss; nonneutropenic infections were more frequent with weekly docetaxel. At least one grade 3–4 toxicity was observed in 38 and 20% (*P*=0.006), 25 and 6% (*P*=0.0003) 20 and 16% (*P*=0.48) of patients as far as any type, haematological or nonhaematological toxicity are considered in the 3-week and weekly arms, respectively. Overall six patients (three in each arm) died during treatment without evidence of disease progression: in the 3-week arm, one (with previous ischaemic hearth disease) died because of atrial fibrillation and subsequent heart failure, one died for septic shock following a broncopneumonitis associated with neutropenia and one died suddenly, with cough and thoracic pain; in the weekly arm, one died with pulmonary thromboembolism and two died for cardiac arrest without other signs of toxicity.

## DISCUSSION

Second-line chemotherapy with docetaxel 75 mg m^−2^ once every 3 weeks has become a standard of treatment for advanced NSCLC patients thanks to two randomised phase III trials (Fossella *et al*, 2000; Shepherd *et al*, 2000). In the first one, chemotherapy with docetaxel 100 or 75 mg m^−2^ was compared to best supportive care, and significantly prolonged median survival (7.0 *vs* 4.6 months). The 100 mg m^−2^ dose was associated with a high toxic death rate, while, at the 75 mg m^−2^ dose, benefits outweighed risks (Shepherd *et al*, 2000). In the second trial, docetaxel 100 and 75 mg m^−2^ were each compared with a control regimen of vinorelbine or ifosfamide. At the 75 mg m^−2^ dose, median time to progression was slightly longer (8.5 *vs* 7.9 months) and a significantly higher proportion of patients were progression-free (17 *vs* 8%) and alive (32 *vs* 19%) at 1 year, as compared with the control arm (Fossella *et al*, 2000). However, myelosuppression associated with docetaxel in both the previous trials was extremely frequent and severe. On these basis, and following a phase II study suggesting weekly docetaxel be less toxic but not less active than the standard schedule (Lilenbaum *et al*, 2001), we performed the DISTAL-1 randomised phase III trial to compare the effects of these two schedules primarily on QOL, and secondarily on overall survival, response rate and toxicity.

In the present study, QoL pattern generally favoured the weekly arm. Advantages in this arm were statistically significant for hair loss, pain and cough. The latter two items clearly show that weekly docetaxel effectively palliated the most frequent symptoms of advanced NSCLC. Diarrhoea was the only QoL item that was unfavourable with the weekly treatment. Nonetheless, although several advantages in toxicity and specific QoL items, no difference in global QoL, measured by questions 29 and 30 of the EORTC C-30 questionnaire, was found. This observation, that we already reported in another trial (Gridelli *et al*, 2003), opens questions regarding sensitivity of the different QoL items, either general or specific, during the course of the disease.

Regardless the great consideration the oncological community has for QoL as a substantial hard end point (American Society of Clinical Oncology, 1996), effects on survival of different treatment options are also crucial for therapeutic decisions. The present study, powered to rule out a 0.67 hazard ratio of death with the weekly schedule, suggests survival be very similar with the two compared schedules (HR of death 1.04), although, of course, interpretation of data in terms of equivalence (Jones *et al*, 1996) is not allowed.

Considering toxicity, our results, consistent with previous ones in phase II (Lilenbaum *et al*, 2001), point out that the weekly schedule is less toxic than the standard 3-weekly one. This holds true for anaemia, leucopenia, neutropenia, febrile neutropenia and alopecia. This information may be of great value, considering the palliative aim of treatment of patients with advanced NSCLC. Differences found in the incidence and severity of neutropenia and febrile neutropenia are also important for a possible reduction of the cost of supportive care (i.v. antibiotics, CSFs administration, hospitalisations) required in such cases. Similar results have been recently reported in a randomised phase II trial of weekly (40 mg m^−2^ for 6 weeks with 2 weeks of rest) and standard (100 mg m^−2^ every 3 weeks) docetaxel in metastatic breast cancer (Tabernero *et al*, 2004). In this study, indeed, overall 49% in the weekly arm *vs* 76% in the 3-weekly arm experienced grade 3–4 adverse events and differences appeared substantial for neutropenia with or without fever, neurotoxicity and stomatitis. All efficacy parameters (response rate, time to progression and survival) were similar in the two groups. Further, similar results have also been reported with paclitaxel, which appears to be even more effective in breast cancer when given weekly instead of 3-weekly (Green *et al*, 2002a, 2002b).

Recently, pemetrexed was found equivalent to 3-week docetaxel, in a large phase III study involving 571 patients in second-line treatment for advanced NSCLC (Hanna *et al*, 2004). In that study, survival, response rate and QOL results were superimposable, but pemetrexed was less toxic particularly for haematological toxicity and related hospitalisations and complications. Based on our results, weekly docetaxel could be at least as interesting as pemetrexed and worth of direct comparison with it.

In conclusion, we suggest that the weekly schedule could be preferred for patients candidate to receive docetaxel as second-line chemotherapy for an advanced NSCLC, because of a better safety profile, some positive effects on QoL and no evidence of strikingly different effect on survival as compared to the 3-week schedule.

As for future perspectives, open questions relate to how to include new drugs, like pemetrexed (Hanna *et al*, 2004), erlotinib (Shepherd *et al*, 2004) or gefitinib (Fukuoka *et al*, 2003), and to whether polychemotherapy can improve the results of single agent treatment. The DISTAL-2 trial will compare weekly docetaxel with combinations of weekly docetaxel plus capecitabine (Nadella *et al*, 2002), vinorelbine (Miller *et al*, 2000; Leu *et al*, 2001) and gemcitabine (Kosmas *et al*, 2001; Spiridonidis *et al*, 2001); for this trial the DISTAL Investigators agreed to apply a slightly modified schedule of docetaxel (i.e. treatment for 3 consecutive weeks followed by 1 week of rest) that should be equivalent to the one studied in the DISTAL-1 trial, but more easy to combine with other cytotoxic agents.

## Appendix. Coauthors and Institutions

Oncologia Medica, Azienda Ospedaliera S Giuseppe Moscati, Avellino (Airoma G, Barzelloni ML, Colantuoni G, Gridelli C, Maione P, Rossi A); Istituto Nazionale Tumori, Napoli: Oncologia Medica B (Barletta E, Formato R, Iaffaioli RV, Pisano C), Unità Operativa Sperimentazioni Cliniche (De Maio E, Di Maio M, Perrone F); Statistica Medica, Seconda Università di Napoli (Gallo C, Signoriello G); Ospedale Monaldi, Napoli: Unità Operativa di Chemioterapia, Day Hospital pneumoncologico, VI Divisione (Battiloro C, Esposito M, Illiano A), Chemioterapia, Day Hospital Pneumologico, VI Divisione, (Brancaccio L, Valentino B); Oncologia, Ospedale Regionale, Parma (Cascinu S^1^, D'Angelo A, Franciosi V, Salvagni S); V Divisione Oncologia Pneumologica, Ospedale Monaldi, Napoli (Lamberti A, Piantedosi FV, Pontillo V); Oncologia Medica, ULSS 15 Regione Veneto, Camposampiero/Cittadella (PD) (Gaion F, Morabito A, Palazzolo G); Oncologia Medica, Ospedale S Chiara, Trento (Arcuri C, Caffo O, Valduga F); Oncologia Medica A, Polo Oncologico, Istituto Regina Elena, Roma (Ceribelli A, Cognetti F, Gelibter A); Oncologia, Ospedali Riuniti, Livorno (Falcone A, Russo F, Tibaldi C); Oncologia Medica, Azienda Ospedaliera Umberto I, Torrette di Ancona (AN) (Boccetti T, Massacesi C, Mazzanti P); Chirurgia Toracica, Ospedali Riuniti, Foggia (Capuano MA, D'Aloia N, Sollitto F); Oncologia Medica, Ospedale Serbelloni, Gorgonzola (MI) (Cullurà D, Isa L, Venezia R); II Pneumologia, Ospedale Marianosanto, Cosenza (Barbera S, Renda F, Volpintesta A); Pneumologia, Ospedale S.Corona, Azienda Ospedaliera S Giuseppe Salvini, Garbagnate (MI) (Rho B, Vaghi A); Oncologia Medica, Ospedale S Paolo, Milano (Foa P, Oldani S); Oncologia Medica, Ospedali Riuniti, Bergamo (Labianca R, Quadri A); Day Hospital Oncologico, Ospedale Civile, Rovereto (TN) (Robbiati SF, Sannicolò M); Divisione di Oncologia Medica ULSS 13, Noale (VE) (Rosetti F, Vinante O); Oncologia Medica, Ospedale Umberto I, Frosinone (Buccilli A, Gamucci T); I Divisione di Pneumologia, Ospedale San Martino, Genova (Felletti R); Oncologia Medica, Ospedale S Bortolo ULSS 6, Vicenza (Schiavon S); Oncologia Medica, Ospedale S Croce, Fano (PS) (Lippe P); Oncologia Medica, Ospedale Civile Madonna del Soccorso, San Benedetto del Tronto (AP) (De Signoribus G); Oncologia Medica, Azienda Ospedaliera di Padova c/o Ospedale Busonera, Padova (Favaretto A); Oncologia Medica, Azienda Ospedaliera G. Salvini, Rho (MI) (Corradini GM); Oncologia, Ospedale S. Giovanni Calibita, Fatebenefratelli, Roma (di Isernia G); Azienda Ospedaliera S Filippo Neri, Roma (Stani S), Cattedra di Oncologia Medica, Dipartimento di Medicina Sperimentale e Patologia, Policlinico Umberto I, Roma (D'Auria G); Servizio di Oncologia, Ospedale C Cantù, Abbiategrasso (MI) (Girmenia G); Centro Catanese di Oncologia, Catania; Day Hospital di Oncologia, Ospedale di Viareggio (LU); Oncologia Medica, Ospedale San Salvatore, L'Aquila; Servizio di Chemioterapia c/o Dipartimento di Oncologia, Policlinico Giaccone, Palermo; Oncologia, Ospedale Civile S. Maria delle Grazie, Pozzuoli (NA); Oncologia Medica e Sperimentale, Istituto Oncologico, Bari; Oncologia Medica, Ospedale Renzetti, Lanciano (CH); Day Hospital Oncoematologico, Divisione di Medicina, Ospedale Civile Umberto I, Nocera Inferiore (SA).

Cesare Gridelli and Francesco Perrone received honoraria from Aventis for participation as speakers in expert panels or boards.
